# A Systematic Review Evaluating the Efficacy of Intra-Ovarian Infusion of Autologous Platelet-Rich Plasma in Patients With Poor Ovarian Reserve or Ovarian Insufficiency

**DOI:** 10.7759/cureus.12037

**Published:** 2020-12-12

**Authors:** Soumya R Panda, Shikha Sachan, Smrutismita Hota

**Affiliations:** 1 Obstetrics and Gynaecology, All India Institute of Medical Sciences, Mangalagiri, Guntur, IND; 2 Obstetrics and Gynaecology, Institute of Medical Sciences, Banaras Hindu University, Varanasi, IND; 3 Radiodiagnosis and Imaging, All India Institute of Medical Sciences, Mangalagiri, Guntur, IND

**Keywords:** decreased ovarian reserve, platelet-rich plasma, premature menopause, premature ovarian insufficiency, prp

## Abstract

The emergence of autologous platelet-rich plasma (PRP) therapy reflects a break-through for infertile patients with premature ovarian failure. To study the efficacy of intra-ovarian infusion of autologous PRP on the improvement of ovarian reserve parameters and the subsequent artificial reproductive technique (ART) cycle outcomes in infertile women with poor ovarian reserve or premature ovarian insufficiency, a systematic search in electronic databases like Medline (through PubMed), Embase, Scopus, Web of Science, and Cochrane was done using relevant search terms. Except for case series, case reports, and review articles, all other types of studies, those evaluated for the effects of intra-ovarian infusion of PRP in subfertile women for decreased ovarian reserve (DOR) or premature ovarian insufficiency (POI) were included in our systematic review. The data were extracted from each eligible study and cross-checked by two authors. Intra-ovarian PRP infusion appears to be effective in ovarian rejuvenation, and the results of the subsequent intracytoplasmic sperm injection (ICSI) cycle are encouraging. PRP intervention was found to be beneficial in terms of an improvement in ovarian reserve parameters (increase in serum anti-mullerian hormone or antral follicle count or decrease in serum follicular stimulating hormone). ICSI cycle performance in terms of the total number of oocytes retrieved, number of two-pronuclei embryos, fertilization rate, number of cleavage stage embryos, number of good quality embryos, and cycle cancellation rate were found to be improved after intra-ovarian PRP infusion as compared to their previous cycle without PRP infusion.

## Introduction and background

A steady decline in the quantity and quality of the oocyte reserves associated with ovarian ageing acts as the principal limiting factor for success in both spontaneous conception and assisted reproductive technology (ART) [1-2]. These aged oocytes are also more prone to errors in deoxyribonucleic acid (DNA) synthesis and cell division, resulting in increased rates of aneuploidy and congenital defects in the resulting conceptions [3]. In the absence of any effective treatment to prevent, delay, or reverse ovarian senescence, various therapeutic strategies like antioxidant dietary supplements containing vitamins C and E, melatonin, dehydroepiandrosterone (DHEA), and coenzyme Q10 have been used to address this issue [4-8]. However, evidence in their support are inconclusive, and their overall effectiveness remains sparse [9-10]. At present, treatment strategies to tackle infertility associated with ovarian insufficiency are commonly in vitro fertilization (IVF) treatment in conjunction with an oocyte donation program or adoption [11-16]. Of late, ovarian function restoration approaches are being investigated rigorously, which can result in healthy, genetically linked offspring in these patients. The successful use of platelet-rich plasma (PRP) in regenerative medicine has led investigators to study its effect in the treatment of conditions like decreased ovarian reserve, premature ovarian failure, etc. [17]. The emergence of autologous PRP therapy reflects a breakthrough approach, showcasing promising results. However, at present, there are very few studies addressing this issue.

This systematic review was conducted to study the effects of intra-ovarian instillation of autologous PRP on ovarian rejuvenation. It was designed to study the efficacy of intra-ovarian infusion of autologous platelet-rich plasma (PRP) on the improvement of ovarian reserve parameters and the subsequent artificial reproductive technique (ART) cycle outcomes in infertile women with poor ovarian reserve or premature ovarian insufficiency.

## Review

Methodology

**Figure 1:**
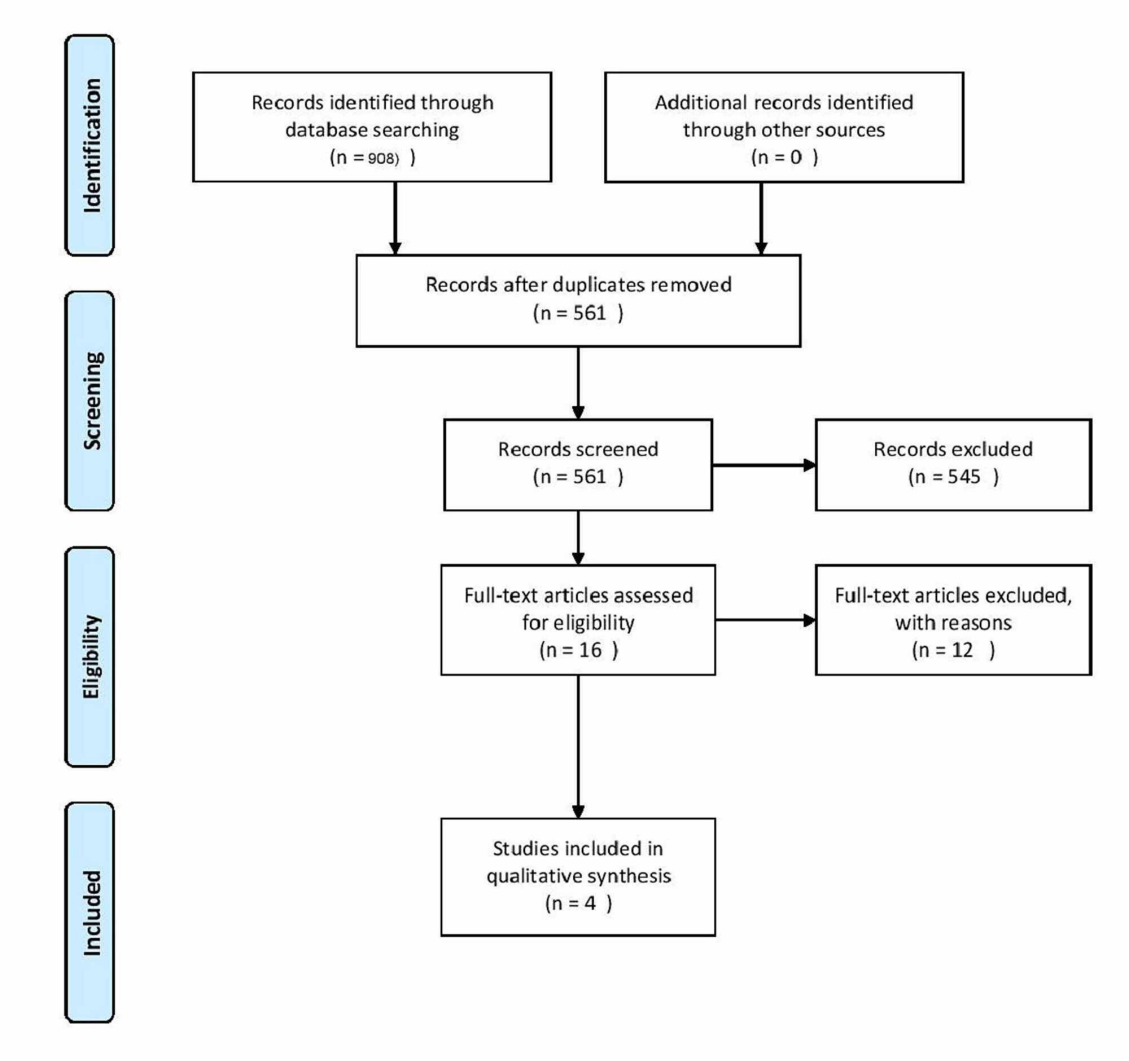
Flow of information through the different phases of the systematic review (according to PRISMA) PRISMA: Preferred Reporting Items for Systematic Reviews and Meta-Analyses

This systematic review was done to assess the effectiveness of intra-ovarian infusion of PRP in sub-fertile women in terms of improvement in ovarian reserve parameters and outcomes after assisted reproduction. We followed the recommendations of the Cochrane Handbook for Systematic Reviews of Interventions and Preferred Reporting Items for Systematic Reviews and Meta-Analyses (PRISMA) guidelines (Figure 1).

A systematic search in electronic databases like Medline (through PubMed), Embase, Scopus, Web of Science, and Cochrane was performed from January 2000 to November 2020. We used search terms like: (“In Vitro Fertilization” OR “IVF” OR “Intracytoplasmic sperm injection” OR “ICSI” OR “Embryo transfer” AND “Platelet-rich plasma” OR “PRP” OR “Autologous platelet-rich plasma” OR “Platelet-rich plasma” and “premature ovarian failure” OR “decreased ovarian reserve” OR “premature menopause” etc). Literature published only in the English language was included in our study.

Inclusion and exclusion criteria

All studies that evaluated the effects of intra-ovarian infusion of PRP in subfertile women for decreased ovarian reserve (DOR) or premature ovarian insufficiency (POI) were included in our systematic review. This review included all types of studies, including case-control studies, cohort studies, or clinical trials. Data regarding ovarian reserve parameters and the intracytoplasmic sperm injection (ICSI)/in vitro fertilization (IVF) cycle characteristics were analyzed in this systematic review. Case series, case reports, and review articles were excluded from our systematic review.

Study selection, data extraction, and quality appraisal

Two authors (S.R.P and S.H.) independently scrutinized the titles and abstracts of the electronic searches according to the predefined eligibility criteria. For relevant studies, full articles were retrieved. The data were extracted from each eligible study and cross-checked by the two authors (S.R.P and S.H.) and by the third author (S.S.). The quality of included studies was assessed based on the criterion provided using the Risk Of Bias In Non-randomized Studies - of Interventions (ROBINS-I) tools for assessing the risk of bias of prospective observational studies [18]. We found four studies to have a low risk of bias. This has been shown in the Appendix.

Meta-analysis

A meta-analysis was planned; however, due to the paucity of available studies and the heterogeneity of the included outcomes, it was deemed inapplicable.

Results

Summary of the Literature Search

The initial electronic literature search yielded 908 publications. After excluding duplicates and irrelevant publications, we found 16 potentially eligible studies. After reading the full text of those 16 articles, 12 articles (three case series, two case reports, one article with insufficient data, one study involving in-vitro experimental design, and five review articles) were excluded. Finally, four studies were included in our systematic review [19-22]. The flow diagram of the literature search and selection of studies is shown in Figure 1.

Study Characteristics

In this systematic review, we have included four studies that evaluated the effectiveness of PRP in infertile women diagnosed with decreased ovarian reserve or premature ovarian failure or menopause. Table 1 outlines the characteristics of all included studies [19-22].

**Table 1:** Characteristics of included studies POI: premature ovarian insufficiency; POR: poor ovarian response; PRP: platelet-rich plasma; FSH: follicle-stimulating hormone; AMH: anti-Müllerian hormone; IVF: in vitro fertilization; USG: ultrasonography; AFC: antral follicle count; ICSI: intracytoplasmic sperm injection; ET: embryo transfer

Sl no.	Study	Country	Study design	Population	Sample size	Intervention	Control	Outcome measures
1.	Cakiroglu et al. 2020 [19].	Turkey	Quasi-experimental-(uncontrolled before and after studies)	Infertile POI	311	Injection of approximately 2-4 ml PRP into each ovary was performed under transvaginal ultrasound guidance.	-	Number of retrieved oocytes, number of mature oocytes, number of 2 pronuclei embryos, fertilization rate, number of cleavage stage embryos
2.	Melo et al. 2020 [20].	Venezuela	Non-randomized clinical trial	Infertile patients planning for IUI/IVF with (i) age 38 years old and above, (ii) baseline FSH, day 3 of the menstrual cycle) > 12 mIU/mL, (iii) AMH < 0.8 ng/mL	Cases-46 Controls-37	200-μL PRP injection received once between days 7 and 9 of the menstrual cycle for three consecutive cycles (cycles 1, 2, and 3).	No intervention	Primary outcome: AFC and serum levels of FSH and AMH as a measure of ovarian reserve. Secondary outcome: number of oocytes collected and fertilization rates during IVF/ICSI; rates of biochemical, clinical, and ongoing pregnancy per participant; and rates of first-trimester miscarriage and live birth
3.	Sfakianoudis et al. 2020 [21].	Greece	Quasi-experimental-(uncontrolled before and after studies)	Four pilot studies were conducted on POR, POI, perimenopause, and menopause	30 subjects for each cohort	Injection of approximately 4 ml PRP into each ovary was performed under transvaginal ultrasound guidance.		AFC, AMH, and oocyte yield in the ICSI-ET cycle, mature metaphase II (MII) oocyte yield in the ICSI-ET cycle post-PRP, number of resulting embryos, and cycle cancellation rate.
4.	Sills et al. 2020 [22].	USA	Quasi-experimental-(uncontrolled before and after studies)	POR with at least one previous failed IVF cycle in perimenopausal or menopausal age	182	1 mL of activated PRP via transvaginal USG guidance		Serum AMH & FSH

Out of four included studies, one was a non-randomized clinical trial and three were quasi-experimental studies (uncontrolled before and after studies). All studies compared the ovarian reserve parameters (AMH, FSH, and antral follicle count (AFC)) before and after PRP infusion. The analysis of the outcome of the ICSI cycle was done in three out of four studies [19-21]. The method of preparation of PRP and time schedule of injection are described in Table 2.

**Table 2:** Method of preparation of platelet-rich plasma and time schedule of injection POI: premature ovarian insufficiency; POR: poor ovarian response; PRP: platelet-rich plasma; FSH: follicle-stimulating hormone; AMH: anti-Müllerian hormone; IVF: in vitro fertilization; AFC: antral follicle count; ICSI: intracytoplasmic sperm injection; ET: embryo transfer

Sl no.	Study	Time schedule of injection	Time schedule of reassessment of ovarian reserve parameters and/or starting of IVF cycle	Method of PRP preparation
1	Cakiroglu et al.2020 [19].	PRP injection was timed randomly in women who were amenorrheic, while in women who reported oligomenorrhea, PRP was injected within 10 days after completion of menstrual bleeding.	On the second menstrual cycle (on the 2^nd^ to 4^th^ day of menses) after the PRP procedure, AFC and serum AMH and FSH levels were re-assessed. Those who were found to have antral follicle(s) at that point were started on controlled ovarian hyperstimulation (COH), while those who did not were followed monthly, up to 6 months, and underwent COH when/if they developed antral follicle(s).	After blood collection, the tubes were centrifuged at 830 g for 8 minutes. Then, a 16 G needle connected to a 5 ml syringe was inserted into the tube and advanced to the buffy coat layer. The PRP was collected by rotating the needle tip. After collecting approximately 2-4 cc PRP from the first tube, the second tube was processed similarly (a total of 4-8 cc PRP was collected). The collected solution was transferred to the re-suspension tube and shaken gently for 30s-1 min.
2	Melo et al. 2020 [20].	Participants who opted for PRP injections received treatment once between days 7 and 9 of the menstrual cycle for three consecutive cycles (cycles 1, 2, and 3).	Following the completion of treatment with PRP, participants were advised to undergo IVF/ICSI, IUI, or timed intercourse as soon as the next menstrual cycle started.	A total of 5 blood collection tubes containing sodium citrate 3.8% were filled with 4.5 mL of blood each and centrifuged at 270g for 10 min. Following centrifugation, 100 μL of the platelet-rich supernatant were transferred from each of 4 of the original blood tubes and mixed with 0.1 mL of 10% calcium chloride. The blood in the remaining fifth tube was not mixed with calcium chloride to allow for quantification of the total number of platelets in the sample. On the day of blood collection (i.e. day 7, 8, or 9 of the cycle), 200 μL of PRP were injected into the cortex of each ovary using a single lumen aspiration needle.
3	Sfakianoudis et al. 2020 [21].	For women presenting with menstrual cycles, such as POR and perimenopausal women PRP administration was done on day-3 of the menstrual cycle. For POI and menopausal women being amenorrheic, PRP administration was performed on a random day.	PRP administration took place during the early follicular phase of the cycle PRP intraovarian infusion treatment was performed at least two months following the last failed ICSI-ET cycle. In the third menstrual cycle post-PRP treatment, all participants received the GnRH antagonist protocol and underwent an ICSI-ET fresh cycle.	Blood samples were collected from the median antebrachial vein. PRP was prepared according to the manufacturer’s instructions employing a RegenACR®-C Kit (Regen Laboratory, Le Mont-sur-Lausanne, Switzerland). Approximately 60 ml of the patient’s peripheral blood was required in order to yield the required volume of PRP. The initial concentration of platelets in peripheral blood was approximately 250,000 platelets/µL. The goal concentration of platelets in PRP was approximately 1,000,000 platelets/µL. According to our protocol, prepared PRP could be stored for one hour at a temperature of 4 ^◦^C if required. However, regarding the vast majority of the participants, PRP intraovarian infusion was performed immediately following preparation.
4	Sills et al. 2020 [22].	All patients had testing for serum AMH, estradiol (E2), and FSH at approximately two-week intervals after ovarian PRP.		Approximately 8-10 mL whole blood was collected by peripheral venipuncture from each patient using a 21G butterfly catheter affixed via vacutainer to negative pressure-receiving tubes (RegenLab; Mont-Sur Lausanne, CH). Samples were immediately labeled and placed in room temperature centrifuge set to 1500g x5 min. Processed blood was then fractionated, and erythrocytes were trapped beneath while lower density components settled atop the separator gel. Less than 3 mL of supernatant (corresponding to relatively platelet-poor plasma fraction) was then aspirated off the top of each column before recapping the vial for gentle tube inversion/resuspension, as per supplier instructions. PRP activation was achieved with calcium gluconate.

Ovarian reserve parameters

*Serum Anti-Mullerian Hormone (AMH)* 

As shown in Table 3, the study conducted by Sfakianoudis et al. [21] showed an increase in serum AMH level after treatment with PRP for the cohorts of poor ovarian response, premature ovarian insufficiency, perimenopause, and menopause, respectively. The difference was found to be statistically significant with p-values of less than <0.0001 in all groups.

**Table 3:** Ovarian reserve parameters FSH: follicle-stimulating hormone; AMH: anti-Müllerian hormone; AFC: antral follicle count

		Cakiroglu et al. 2020 [19]. (N=311)	Melo et al 2020 [20]. (N=46)	Sfakianoudis et al.2020 [21]. (pilot of poor ovarian response) (N=30)	Sfakianoudis et al., 2020 [21]. (premature ovarian insufficiency pilot) (N=30)	Sfakianoudis et al. 2020 [21]. (perimenopause pilot) (N=29)	Sfakianoudis et al. 2020 [21]. (menopause pilot) (N=25)	Sills et al. 2020 [22]. (N=182)
AMH (ng/mL)	Pretreatment	0.13 ± 0.16	0.62^*^ (0.47 to 0.76)	0.66 ± 0.20	0.168 ± 0.04	0.94 ± 0.29	0.12 ± 0.04	0.18 ± 0.25
	Posttreatment	0.18 ± 0.18	1.01^*^ (0.9 to 1.3)	1.14 ± 0.26	0.57 ± 0.05	1.26 ± 0.26	0.40 ± 0.13	0.24 ± 0.05
	p-value	<0.01	<0.001	<0.0001	<0.0001	<0.0001	<0.0001	0.0016
FSH (mIU/L)	Pretreatment	41.9 ± 24.7	13.6 ^*^ (12.9 to 17.5)	10.71 ± 1.62	49.82 ± 6.19	18.47 ± 2.47	80.69 ± 5.61	52.67 ± 4.64
	Posttreatment	41.6 ± 24.7	9.07^*^ (8.3 to 10.5)	8.95 ± 1.40	36.16 ± 6.6	15.85 ± 3.69	48.03 ± 5.90	64.68 ± 5.5
	p-value	p=0.87	<0.001	0.1342	<0.0001	0.0024	<0.0001	<0.0001
Total AFC (n)	Pretreatment	0.5 ± 0.5	4^*^ (3 to 5)	2.63 ± 0.93	0 ± 0	1.43 ± 0.55	0 ± 0	
	Posttreatment	1.7 ± 1.4	7^*^ (6 to 8)	5.20 ± 1.35	1.39±0.37	3.64 ±0.78	1.23 ± 0.46	
	p-value	<0.01	< 0.001	<0.0001	<0.0001	<0.0001	<0.0001	

Similarly, Melo et al. found an increase in the median value of the post-treatment level of serum AMH (in ng/ml) from the respective pretreatment level [20]. The median difference was 0.5 (0.43 to 0.57), and the increase in serum AMH with PRP infusion was found to be statistically significant (p-value <0.001). This has been depicted in Table 3.

At the same time in the study by Cakiroglu et al. [19] and Sills et al. [22], the level of serum AMH was also found to be increased (Table 3) after intra-ovarian PRP infusion with a statistically significant difference (p-values of <0.01 and 0.0016 for the respective studies). Sills et al. [22] found platelet count as a parameter likely to predict AMH response to intra-ovarian PRP injection.

Serum Follicular-Stimulating Hormone (FSH)

In the study by Sfakianoudis et al., the serum FSH levels were decreased post-treatment with PRP for all the studied cohorts [21]. Except for the cohort of women with poor ovarian reserve (p-value=0.1342), all other cohorts were having a statistically significant decrease in serum FSH level (p-values of <0.0001, 0.0024, and <0.0001 for cohorts of premature ovarian insufficiency, perimenopause, and menopause respectively).

Melo et al. found a decrease in the median of post-treatment values of serum FSH with a median difference of − 5.5 (− 6.3 to − 4.9) and the difference was found to be statistically significant ( p-value -0.001) [20].

In the study by Cakiroglu et al., the average of pretreatment and post-treatment values of serum FSH (all values in mIU/ml) were found to be 41.9 ± 24.7 and 41.6 ± 24.7 [19]. But this decrease in serum FSH levels was not statistically significant (p-value=0.87).

Moreover, Sills et al. found an increase in the post-treatment serum FSH value of 64.68 ± 5.5 from its pretreatment value of 52.67 ± 4.64 with a statistically significant difference (p-value<0.0001) [22].

These findings are tabulated in Table 3.

Antral Follicle Count (AFC)

As far as antral follicle count is concerned, the studies by Sfakianoudis et al. [21] (in all of their studied cohorts) and Melo et al. [20] found an increase in AFC after treatment with PRP, and this increase was statistically extremely significant with p-values of <0.0001 in each of the studies. This is shown in Table 3.

Again in the study by Cakiroglu et al. [19], the average of pretreatment and post-treatment values of antral follicle count were found to be 0.5 ± 0.5 and 1.7 ± 1.4 indicating a statistically significant increase (p-value= <0.01).

Outcomes of the ICSI Cycle

Sfakianoudis et al. compared the post-PRP ICSI cycle with the prior ICSI cycle as control [21]. They found the average of total number of retrieved oocytes (3.37 ± 1.54 versus 1.20 ± 0.76; p-value-<0.0001), number of mature oocytes (2.97 ± 1.38 versus1.00 ± 0.79; p-value-<0.0001), number of two-pronuclei embryos (2.43 ± 1.38 versus 0.73 ± 0.52; p-value-<0.0001), number of cleavage stage embryos (1.93 ± 1.26 versus 0.60 ± 0.56; p-value-<0.0001), and cancellation rate (30% versus 63.3%; p-value 0.0191) were better in the post-PRP ICSI cycle as compared to the control, which bore a statistically significant difference. The number of good quality embryos produced was also more in the post-PRP ICSI cycle but this difference was not statistically significant when compared with the controls.

Similarly in the study by Melo et al., the total number of retrieved oocytes (median value of 5.0 ranging from 2.0 to 9.0 versus the median value of 3.0 with a range of 0.0-6.0; p-value-<0.001), fertilization rate (median value of 0.5 ranging from 0.33-1.0 versus median value 0.5 ranging from 0.0-1.0; p-value <0.0001), and the number of good quality embryos (22 /100 versus 6 /55; p-value-<0.0001) were better in the post-PRP ICSI cycle compared to the control which bore a statistically significant difference (Table 4) [20].

**Table 4:** Outcomes of the ICSI cycle ICSI: intracytoplasmic sperm injection; PRP: platelet-rich plasma

Sl no.	Study	Participants	Number of retrieved oocytes	Number of mature oocytes	Number of 2 pronuclei embryos	Fertilization rate	Number of cleavage stage embryos	Good quality embryo (grade-1&2)	Cancellation Rate
	Cakiroglu et al., 2020 [19].	-	1.81 ± 1.30 (N=100)	1.47 ± 0.76 (N=93)	1.24 ± 0.49 (N=82)	55.8 ± 29.1 (N=82)	1.18 ± 0.39 (N=82)	-	-
	Melo et al., 2020 [20].	Cases (PRP infusion) N=22	5.0 (2.0–9.0) (N=22)	-	-	0.5 (0.33–1.0)	-	22 (100)	-
		Controls (N=18)	3.0 (0.0–6.0)	-	-	0.5 (0.0–1.0)	-	6 (55)	-
		P-Value	< 0.001	-	-	0.51	-	0.03	-
	Sfakianoudis et al., 2020 [21].	Cases (Post-PRP ICSI Cycle)	3.37 ± 1.54	2.97 ± 1.38	2.43 ± 1.38	-	1.93 ± 1.26	28/58 (48.2%)	9/30 (30%)
		Controls (Prior ICSI cycle)	1.20 ± 0.76	1.00 ± 0.79	0.73 ± 0.52	-	0.60 ± 0.56	8/18 (44.4%)	19/30 (63.3%)
		P-Value	< 0.0001	< 0.0001	< 0.0001	-	< 0.0001	0.7945	0.0191

In the study by Cakiroglu et al., ART was attempted in 201 women who had at least one antral follicle after PRP out of which oocyte retrieval was performed in 130 (64.7% were stimulated) women [19]. Out of this, in 82 women (40.8% of stimulated cycles), at least one cleavage-stage embryo was obtained and embryo cryopreservation or fresh embryo transfer was performed. These embryos were grade 1/2 morphologically. The mean number of oocytes per retrieval was 1.81 ± 1.30. The mean numbers of two-pronuclei (2 PN) and cleavage-stage embryos obtained in women who developed embryos were 1.24 ± 0.49 and 1.18 ± 0.39, respectively. Among the 82 women who developed embryos, 25 preferred to store cryopreserved embryos for transfer at a later stage and 57 underwent embryo transfer. Of those who underwent embryo transfer, 28/57 (49.1%) were fresh embryo transfers and 29/57 (50.9%) were frozen-thawed embryo transfers; 7/28 (25.0%) of fresh embryo transfers and 6/29 (20.7%) of frozen-thawed embryo transfers resulted in a pregnancy (Table 4).

Discussion

This systematic review involved the data analysis of 663 subfertile women who were intervened with an intra-ovarian infusion of PRP from four studies. PRP intervention was found to be beneficial in terms of improvement in ovarian reserve parameters (increase in serum AMH or antral follicle count or decrease in serum FSH). The outcome of the ICSI cycle was studied in three out of four included studies. The outcome of the ICSI cycle in terms of the total number of oocytes retrieved, number of two-pronuclei embryos, fertilization rate, number of cleavage stage embryos, number of good quality embryos, and cycle cancellation rate were found to be improved after the intra-ovarian PRP infusion as compared to their previous cycle without PRP infusion.

A study by Cakiroglu et al. found that women who did not have an antral follicle at the time of PRP injection were less likely to respond to treatment as compared to those who had one or two antral follicles [19]. Similarly, women with the lowest quartile for serum AMH and the highest for serum FSH were less likely to respond. The final conclusion for the same study was that PRP helps activate existing preantral and/or early antral follicles and that the number of remaining follicles in the ovaries of women with POI likely determines the extent of their response [19]. However, at this point in time, we cannot generalize this finding and there is an obvious need for future well-controlled studies to identify the subpopulation that can get the maximum benefit from PRP infusion.

Many studies have documented that the use of PRP can reduce the features of inflammation, postoperative blood loss, infection, and narcotic requirements. Also, PRP has a role in the acceleration of osteogenesis and wound and soft tissue healing [23-24]. The granules in platelets contain certain important growth factors such as transforming growth factor-β, insulin-like growth factors 1 and 2 (IGF-1 and IGF-2), vascular endothelial growth factor (VEGF), epidermal growth factor (EGF), basic fibroblast growth factor, and hepatocyte growth factor (HGF), which forms the basis for the tissue regenerative properties of PRP [17,25-26].

These are the same growth factors that are considered to be vital for cell migration and differentiation, as well as for proliferation, activation of angiogenesis, and tissue regeneration [27-28]. Of note, studies have found an inverse relationship between aging and concentrations of growth hormone and IGF-1 [29].

Specifically, the application of PRP in ovarian rejuvenation has not been studied in detail. Till now, only a few studies have addressed this issue [30]. In their study, Bakacak et al. found that PRP can have a significant effect on preventing ischemia and reperfusion damage in rats following bilateral adnexal torsion and surgical detorsion [31]. They concluded that this action was mainly through an increase in VEGF. Few studies in the form of case series got encouraging results after evaluating the application of PRP for managing a thin endometrium, recurrent implantation failure, and poor response to controlled ovarian stimulation [32-34].

A case was reported documenting a biochemical pregnancy and subsequent miscarriage following autologous PRP intra-ovarian infusion in an infertile woman with premature ovarian failure [35]. Similarly, cases documenting live birth in poor responders following PRP infusion have also been reported [32]. Additionally, other studies support a contribution of PRP treatment to follicular growth and maturation [36].

Similar to our study, recently, a few case series also highlighted the efficacy of intra-ovarian PRP injection. In the case series described by Pantos et al. [37], Sills et al. [38], and Sfakianoudis et al. [32], the pretreatment serum AMH was increased after the intra-ovarian infusion of PRP. However, this increase in serum AMH levels was statistically significant only in the study by Pantos et al. [37] (p-value 0.0395), and in the studies by Sfakianoudis et al. [32] and Sills et al. [38], the difference was statistically not significant (p-values 0.4546 and 0.17, respectively).

Similarly, in these case series, the pretreatment serum FSH values were decreased after the intra-ovarian infusion of PRP [32,37-38]. This decrease was found to be statistically significant in all of the above studies with p-values of 0.0310, 0.01, and 0.0053, respectively.

In their case series, Pantos et al. found a post-treatment increase in the mean values of AFC (0 ±0 and 2 ± 1.41, respectively) [37]. However, this was not found to be statistically significant with a p-value of 0.0699.

Strengths and limitations

This is a systematic review evaluating the effect of intra-ovarian infusion of PRP on ovarian reserve parameters and ICSI cycle performance in women with decreased ovarian reserve or premature ovarian failure. However, some limitations should be considered in the interpretation of this systematic review. The less number of studies (n = 4) and lack of homogeneity among the included studies is the foremost limitation. This is the reason that we could not perform a meta-analysis. In most of the studies, pregnancy characteristics, such as clinical pregnancy rate, miscarriage rate, chemical pregnancy rate, and live birth rate have not been evaluated. Only a few studies have evaluated the ICSI cycle performance. Lastly, most of the included studies are quasi-experimental studies and not a single RCT is included. However, as the intra-ovarian infusion of PRP is a rare and the newest form of therapy in the field of infertility, such limitations are expected while conducting a systematic review. Moreover, the encouraging result of our study has paved the way for conducting future, well-organized, randomized controlled trials.

## Conclusions

Our systematic review showed that intra-ovarian autologous PRP infusion increases the ovarian reserve parameters resulting in increased mature oocyte yield, fertilization rate, as well as the formation of good-quality embryos. Thus, this sensational novel therapy is particularly a great finding in the field of reproductive medicine, as this has the potential to put a full stop to our long search for the question of poor ovarian reserve and getting a genetically linked baby. Definitely, there is a great need for future, high-quality randomized controlled trials to estimate its efficacy in terms of clinical pregnancy and live birth rate. Also, there is a need to identify an optimum level of serum AMH or another marker of ovarian reserve for the success of intra-ovarian PRP infusion and identify the subpopulation that would get the most benefit from PRP.

## Appendices

**Table 5:** Results of critical appraisal

Study Domain	1 Confounding Domain	2 Selection of Participants Domain	3 Classification of interventions Domain	4 Departure From intended interventions Domain	5 Missing Data Domain	6 Measurements of Outcome Domain	7 Selection of Reported Results	Overall Judgment	
Cakiroglu 2020 [19]	Low risk	Low risk	Low risk	Low risk	Low risk	Low risk	Low risk	Low risk	
Melo 2020 [20]	Low risk	Low risk	Low risk	Low risk	Low risk	Low risk	Low risk	Low risk	
Sfakianoudis 2020 [21]	Low risk	Low risk	Low risk	Low risk	Low risk	Low risk	Low risk	Low risk	
Sills et al. 2020 [22]	Low risk	Low risk	Low risk	Low risk	Low risk	Low risk	Low risk	Low risk	
